# Supplementary material for: MetaRibo-Seq measures translation in microbiomes
Source: Nat Commun. 2020 Jun 29;11:3268. doi: 10.1038/s41467-020-17081-z (PMC7324362; doi:10.1038/s41467-020-17081-z)
Supplement: Supplementary file 10 — Supplementary Data 7 [file 41467_2020_17081_MOESM10_ESM.zip › File2/Confidence_High_Taxonomy/264729_out.krona.html]

Javascript must be enabled to view this page.

members
magnitude
magnitudeUnassigned
count
unassigned
taxon
rank

264729\_out

10

superkingdom
2
10

10
phylum
976

class
200643
10

10
171549
order

10
171550
family

genus
239759
10

species
328814
1

SRS048060\_contig\_number\_7367

species
1930002

SRS146813\_contig\_number\_18264
1

2

SRS065397\_contig\_number\_contig-100\_2003.83730SRS1054691\_contig\_number\_11052
species
1465754

1816676
species
4

SRS018656\_contig\_number\_188SRS045826\_contig\_number\_22617SRS142599\_contig\_number\_26049SRS146888\_contig\_number\_10345

species
1946007

SRS1041140\_contig\_number\_1461
1


SRS143523\_contig\_number\_15039
1
species
1118061
